# Case report: immunotherapy response in pancreatic cancer after MSI-H emerged post-chemotherapy

**DOI:** 10.3389/fonc.2026.1659656

**Published:** 2026-04-13

**Authors:** Kareem Gharaybeh, Karim Khalidi, Sana Al-Sukhun

**Affiliations:** 1 Stratford Preparatory High School, San Jose, San Jose, CA, United States; 2 Al Khalidi Hospital and Medical Center, Amman, Jordan; 3 Al Hyatt Oncology Practice., Amman, Jordan

**Keywords:** checkpoint inhibitors, immunotherapy, liquid biopsy, microsatellite instability-high (MSI-H), molecular profiling, pancreatic ductal adenocarcinoma (PDAC)

## Abstract

**Background:**

Microsatellite instability-high (MSI-H) is an established biomarker of response to immune checkpoint inhibitors, though it is rarely observed in pancreatic ductal adenocarcinoma (PDAC). Even less common is the emergence of MSI-H during the disease course, particularly when identified through liquid biopsy. Such findings raise important considerations regarding tumor evolution, assay concordance, and therapeutic decision-making.

**Case Presentation:**

We report the case of a 76-year-old woman with borderline resectable PDAC who received first-line chemotherapy for nearly 20 months, maintaining excellent performance status and disease control. Upon clinical and biochemical progression, liquid biopsy using Guardant360 CDx revealed MSI-H status and a TP53 H214fs mutation, both absent in her initial molecular profile. A concurrent FoundationOne^®^ Liquid CDx analysis detected a high circulating tumor DNA fraction but reported the tumor as microsatellite stable, highlighting a discordance in MSI classification across platforms. Given her favorable clinical condition and emerging molecular findings, pembrolizumab was initiated. The patient experienced sustained clinical benefit and radiographic stability for 13 months before progressing with peritoneal carcinomatosis and ascites.

**Conclusions:**

This case illustrates the potential emergence of MSI-H during the disease course of PDAC and highlights the clinical value of repeat molecular profiling at progression. It also highlights clinically relevant discrepancies in MSI detection between liquid biopsy platforms and supports the role of immunotherapy in selected patients with evolving molecular features.

## Introduction

Pancreatic ductal adenocarcinoma (PDAC) is one of the most lethal malignancies worldwide, with a five-year survival rate under 10% and a rising global burden that is projected to make it the second leading cause of cancer-related death by 2030 (1). Most patients present with advanced or unresectable disease, and although front-line chemotherapy regimens have modestly extended survival, long-term disease control remains elusive. Only 30–50% of patients are eligible for second-line therapy due to rapid performance status deterioration (2, 3). Even among those who do receive additional therapy, responses are often limited to disease stabilization (4).

Incorporating genomic profiling into PDAC management has helped identify a small subset of patients with targetable alterations. However, fewer than 25% of PDAC tumors harbor actionable molecular changes (5). One such alteration is microsatellite instability-high (MSI-H) or mismatch repair deficiency (dMMR), which occurs in approximately 1–2% of PDAC cases and predicts a favorable response to immune checkpoint inhibitors such as pembrolizumab (6, 7). Despite these advances, the application of immunotherapy in PDAC remains limited due to its immunosuppressive microenvironment and typically low neoantigen load (8).

We present a rare case of borderline resectable PDAC in a patient whose tumor evolved from microsatellite stable (MSS) to MSI-H following nearly 20 months of chemotherapy. This molecular change was identified by liquid biopsy via Guardant360 CDx, while a concurrent FoundationOne^®^ Liquid CDx test reported the tumor as MSS, highlighting inter-assay discordance. The patient subsequently experienced a durable clinical response to pembrolizumab. This case highlights the relevance of re-profiling at progression and raises important questions regarding tumor evolution, molecular diagnostics, and precision immunotherapy in PDAC.

## Case report

A 76-year-old woman presented with obstructive jaundice and epigastric pain. Her past medical history was otherwise unremarkable, and there was no known family history of pancreatic or Lynch-associated malignancies. Baseline performance status was ECOG 1. At presentation the patient reported epigastric pain and jaundice. Physical examination revealed mild right upper quadrant tenderness without palpable masses. Laboratory evaluation demonstrated elevated bilirubin consistent with biliary obstruction. Contrast-enhanced CT imaging demonstrated a mass in the pancreatic head encasing the superior mesenteric vein. Fine needle aspiration confirmed pancreatic adenocarcinoma. Liquid biopsy using Guardant360 detected KIT amplification. The tumor was initially microsatellite stable. At disease progression, repeat genomic profiling revealed the emergence of MSI-H status. No pathogenic variants were detected in MLH1, MSH2, MSH6, PMS2, or EPCAM. Initial treatment consisted of modified FOLFIRINOX chemotherapy. Following treatment intolerance and detection of KIT amplification on molecular profiling, targeted therapy with imatinib was initiated. Capecitabine was subsequently added in a metronomic schedule to improve disease control while maintaining tolerability (9). She maintained excellent performance status throughout nearly 20 months of treatment, during which her disease remained clinically and radiographically stable. Subsequently, she developed mild epigastric discomfort, and restaging imaging revealed disease progression, accompanied by an elevation in serum CA 19–9 levels. Repeat biopsy was considered at the time of progression; however, the patient declined additional invasive procedures due to her age and preference to avoid further interventions.

To guide subsequent treatment, comprehensive genomic profiling was performed using a liquid biopsy (Guardant360 CDx), which employed next-generation sequencing of circulating cell-free DNA. This analysis identified a microsatellite instability-high (MSI-H) phenotype and a TP53 H214fs frameshift mutation. These findings differed from her baseline molecular profiling—also conducted via Guardant360 prior to systemic therapy—which had reported microsatellite stability and no TP53 alteration. To further evaluate this discrepancy, an additional ctDNA analysis was conducted using FoundationOne^®^ Liquid CDx, four days after the Guardant360 CDx test, during the same clinical progression assessment. No systemic therapy was administered between the two blood draws, making clinically meaningful genomic evolution over this short interval unlikely. Despite a high tumor fraction, this assay classified the tumor as microsatellite stable, highlighting discordance in MSI status between liquid biopsy platforms. The FoundationOne^®^ Liquid CDx report also showed a blood tumor mutational burden of 10 mutations/Mb and a DNMT3A R882C variant consistent with clonal hematopoiesis, with an estimated circulating tumor fraction of approximately 10%.

Given the detection of MSI-H by a validated plasma assay, the established sensitivity of MSI-H tumors to PD-1 blockade, and the patient’s preference to avoid further cytotoxic chemotherapy, pembrolizumab was selected as the next therapeutic strategy. This decision was made through a shared decision-making discussion with the patient. At a three-week follow-up visit, the patient reported improved well-being, and a reduction in CA 19–9 levels was noted. Follow-up imaging at three months demonstrated stable disease, with no evidence of metastatic progression.

**Figure 1:**
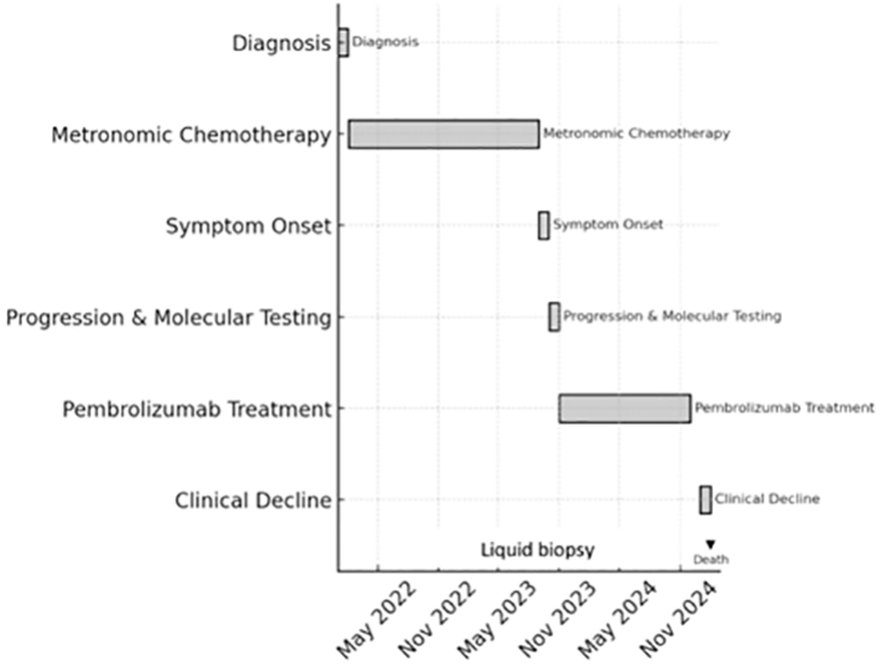
Timeline of the patient’s clinical course and corresponding serum CA 19–9 levels, including diagnosis of borderline resectable PDAC, first-line chemotherapy, symptomatic progression, molecular profiling, initiation of pembrolizumab, biochemical response (CA 19–9 decline from 380 U/mL to 5 U/mL), and eventual disease progression with peritoneal carcinomatosis.

She continued on pembrolizumab for an additional 13 months with stable disease confirmed on imaging performed every three months. Despite the development of mild recurrent epigastric discomfort, she opted to continue therapy, prioritizing quality of life in the absence of treatment-related toxicity. Indeed, she did not develop any clinically significant immune-related adverse events and did not require treatment interruption or systemic immunosuppression during 13 months of pembrolizumab therapy. Over the following two months, she experienced clinical and radiologic progression marked by peritoneal carcinomatosis and ascites, and ultimately passed away from disease progression. A visual summary of the patient’s diagnostic, therapeutic, and disease progression milestones is presented in **Figure 1**.

The patient provided written informed consent for the use of de-identified clinical, molecular, and imaging data from her medical records for the purposes of this publication.

## Discussion

### Acquired MSI-H in PDAC: a rare phenomenon

Pancreatic ductal adenocarcinoma (PDAC) is an aggressive malignancy with limited effective therapies and poor survival outcomes. While immunotherapy has revolutionized treatment in several cancers, its benefit in PDAC remains marginal due to the tumor’s low neoantigen burden, limited T-cell infiltration, and immunosuppressive microenvironment (6, 7). Nonetheless, a small subset (~1–2%) of PDAC cases exhibit microsatellite instability-high (MSI-H) or mismatch repair deficiency (dMMR), which are established biomarkers for response to immune checkpoint blockade (ICB) (5, 8).

The earlier therapeutic phase of this patient’s disease course has been previously reported (9).The current report extends these observations by documenting subsequent molecular evolution with emergence of MSI-H and response to immune checkpoint blockade. In this case, MSI-H status was not detected at baseline but emerged after nearly 20 months of chemotherapy, supporting the possibility of an acquired somatic event rather than a hereditary MMR defect and suggesting possible tumor evolution under treatment pressure. Evidence of dynamic clonal changes is further supported by molecular differences observed across serial liquid biopsies: a KIT amplification detected in the baseline Guardant360 analysis was no longer present at the time of progression. Notably, this alteration had previously been clinically relevant, guiding a prolonged therapeutic response to imatinib in this patient, as reported by our group (9). Neither plasma-based assay detected pathogenic alterations in MLH1, MSH2, MSH6, PMS2, or EPCAM. The patient had no personal or family history suggestive of Lynch syndrome or other hereditary cancer syndromes. Germline testing was offered but declined.

We acknowledge that lack of tissue confirmation and functional mismatch repair testing constrains diagnostic certainty. However, increasing evidence indicates that MSI testing may be a more accurate predictor of immunotherapy benefit than MMR-IHC, because MSS tumors, generally marked by low mutational burden, produce too few neoantigens to drive an effective ICB response, even when MMR protein loss is detected by IHC (10). Others have demonstrated systemic chemotherapy, particularly alkylating agents, can induce measurable genomic instability—manifesting as MSI, LOH, and reduced expression of key mismatch-repair proteins such as MLH1 and MSH2 (11). Chemotherapy-related genomic stress appears to play a broader role across solid tumors, with studies showing that a substantial proportion of patients develop treatment-associated MSI and LOH—particularly when mismatch-repair proteins such as MLH1 or MSH2 are suppressed—supporting the concept that cytotoxic therapy can transiently or persistently shift an originally MSS tumor toward an unstable phenotype. Additional mechanistic support comes from temozolomide-treated gliomas, where recurrent tumors frequently acquire new MSH6 or MSH2 loss-of-function mutations, illustrating how sustained DNA damage can generate secondary MMR deficiency (12). Collectively, these data outline a coherent evolutionary model; prolonged genotoxic therapy imposes selective pressure and genomic stress that can either unmask pre-existing MMR-deficient subclones or induce new MMR pathway defects. This model provides a credible explanation for the rare but clinically important scenario in which initially MSS tumors are later found to be MSI-H.

### Inter-assay discordance in ctDNA-based MSI detection

A second important feature of this case is the discordance in MSI status between two well-validated circulating tumor DNA (ctDNA) assays. Guardant360 CDx identified MSI-H and a TP53 H214fs mutation, while FoundationOne^®^ Liquid CDx, performed on a separate blood draw obtained four days later during the same clinical progression assessment, reported microsatellite stability despite a high circulating tumor DNA fraction. This discrepancy reflects differences in assay design, such as the number and type of microsatellite loci evaluated, bioinformatic algorithms, and reporting thresholds (13). Guardant360 uses proprietary algorithms across a large panel of microsatellite loci, while FoundationOne relies on a different set and analysis approach. Factors such as ctDNA yield, tumor heterogeneity, and sampling timing may also contribute to discordant findings (13). While a technical artifact remains possible, particularly in the setting of discordant MSI results from two plasma assays, the emergence of a new TP53 frameshift mutation together with the subsequent durable clinical benefit from pembrolizumab makes a purely technical explanation less likely.

Importantly, this was not a tissue-versus-liquid discordance. Both assays were plasma-based and obtained during the same clinical progression assessment, reducing the likelihood that the discrepancy reflects spatial sampling bias or clinically meaningful temporal evolution. Given the patient’s subsequent durable clinical benefit from pembrolizumab, the Guardant-detected MSI-H finding appears to be biologically and clinically relevant. This case emphasizes the need for confirmatory testing or clinical correlation when molecular results from ctDNA are used to guide treatment.

### Therapeutic response and clinical implications

After identification of MSI-H, the patient initiated pembrolizumab and maintained stable disease for 13 months, with good quality of life and minimal toxicity. This durable response is consistent with results from the KEYNOTE-158 trial, where pembrolizumab demonstrated clinical activity in MSI-H/dMMR non-colorectal tumors, including PDAC (5) chemotherapy—can still confer responsiveness to immunotherapy.

We acknowledge that causality cannot be definitively established in this setting, considering the absence of functional mismatch repair testing and repeated tissue sampling. However, we interpret the response in light of an FDA-approved plasma assay calling MSI-H, a concordant clinical and biochemical response, and known class activity of PD-1 blockade in MSI-H/dMMR malignancies. While plasma-based MSI detection has recognized technical limitations and may be influenced by circulating tumor DNA fraction and assay-specific analytical thresholds, the molecular findings in this case were interpreted in the context of the longitudinal clinical course and treatment response.

This report builds upon the patient’s previously documented durable response to front-line metronomic chemotherapy (9). Together, the two treatment phases illustrate an evolving tumor biology that transitioned from cytotoxic sensitivity to immune responsiveness, reinforcing the importance of longitudinal molecular assessment in guiding treatment.

### Practice and research considerations

Key takeaways from this case include:

- Re-testing at disease progression may uncover actionable changes, such as MSI-H, that were absent at diagnosis.
- Discrepancies between ctDNA assays—even when technically robust—warrant cautious interpretation, particularly for biomarkers with therapeutic implications.
- Mechanistic studies are needed to elucidate whether acquired MSI-H arises through clonal evolution, mismatch repair disruption, or chemotherapy-induced genomic stress.
- Efforts to harmonize MSI testing methodologies across platforms will improve clinical decision-making and patient selection for immunotherapy.

## Conclusion

This case highlights the rare phenomenon of acquired MSI-H in pancreatic cancer following prolonged chemotherapy, supporting the possibility of an acquired somatic event rather than a hereditary mismatch-repair defect and suggesting tumor evolution under treatment pressure. It underscores the importance of re-evaluating tumor molecular profiles at progression and the need for cautious interpretation of liquid biopsy assays, especially when platform discordance exists. Dynamic changes in tumor biology, even in traditionally immunotherapy-resistant cancers such as PDAC, may present new therapeutic opportunities and support personalized, evolving treatment strategies.

## Data Availability

The original contributions presented in the study are included in the article/supplementary material. Further inquiries can be directed to the corresponding author.
